# Neutralizing Antibody Response, Safety, and Efficacy of mRNA COVID-19 Vaccines in Pediatric Patients with Inflammatory Bowel Disease: A Prospective Multicenter Case—Control Study

**DOI:** 10.3390/vaccines10081265

**Published:** 2022-08-06

**Authors:** Kyung Jae Lee, So Yoon Choi, Yoo Min Lee, Han Wool Kim

**Affiliations:** 1 Department of Pediatrics, Hallym University Sacred Heart Hospital, Anyang 14068, Korea; 2 Department of Pediatrics, College of Medicine, Hallym University, Chuncheon 24252, Korea; 3 Department of Pediatrics, Seoul National University Children’s Hospital, Seoul 03080, Korea; 4 Department of Pediatrics, Kosin University Gospel Hospital, Kosin University College of Medicine, Busan 49267, Korea; 5 Department of Pediatrics, Soonchunhyang University Bucheon Hospital, Soonchunhyang University College of Medicine, Bucheon 14584, Korea

**Keywords:** COVID-19 vaccines, COVID-19 breakthrough infections, inflammatory bowel diseases, adolescent, pediatric

## Abstract

The vaccination of immunocompromised children against coronavirus disease 2019 is an important public health issue. We evaluated the serological response, safety, and efficacy of the BNT162b2 vaccine in children with and without inflammatory bowel disease (IBD). A prospective, multicenter, case–control study was conducted in a pediatric population, including patients with IBD, aged 12–18 years. Clinical characteristics, safety profile, and serum samples for surrogate virus-neutralizing antibody testing pre- and post-BNT162b2 vaccination were assessed. The breakthrough infection rate during the Omicron outbreak was calculated to evaluate efficacy. Fifteen controls and twenty-three patients with IBD were enrolled. After two vaccine doses, the median level of percentage inhibition was highly increased, without significant differences between the groups (control 96.9 and IBD 96.3). However, it was significantly reduced in IBD patients receiving combination therapy (anti-tumor necrosis factor-α + immunomodulators) relative to those in other therapies and controls. Serious adverse events were not observed. The breakthrough infection rate was 42.1%, without statistical differences between the groups. Immunization with BNT162b2 in patients with IBD was comparable with that in healthy adolescents in terms of immunogenicity and safety. Nevertheless, the efficacy of BNT162b2 in preventing infection caused by the Omicron variant in the pediatric population was insufficient.

## 1. Introduction

The coronavirus disease 2019 (COVID-19), caused by severe acute respiratory syndrome coronavirus 2 (SARS-CoV-2), has been a global health problem since December 2019 [1]. COVID-19 has spread quickly worldwide, and vaccinations against SARS-CoV-2 have been approved after numerous scientific efforts [2]. Currently, major countries recommend that children aged 5 years and above with comorbidities who are at risk of severe COVID-19 should receive vaccination, and now consider vaccinating healthy children as part of the national vaccination strategies [3,4,5]. 

The incidence of inflammatory bowel diseases (IBDs), including Crohn’s disease (CD) and ulcerative colitis (UC), has increased notably with rapid urbanization in Asia [6,7], especially in Korea, and approximately 25% of IBD patients are diagnosed in pediatric ages before the age of 20 [8]. IBDs are immune-mediated chronic inflammatory diseases, and patients with IBD need to take prolonged immunosuppressive drugs, such as prednisolone, immunomodulators (e.g., azathioprine (AZP), methotrexate (MTX), and calcineurin agents), and/or biologic agents (e.g., infliximab, adalimumab, golimumab, and vedolizumab) [9,10]. Furthermore, approximately half of Korean pediatric CD patients have perianal fistulas and/or abscesses at the time of diagnosis, which requires more potent immunosuppressants, such as anti-tumor necrosis factor-α (anti-TNF) agents [7,10]. Although the introduction of immunosuppressants including biologics is an effective treatment strategy, it can increase the susceptibility to infection and malignant diseases [11,12]. Furthermore, most immunosuppressive drugs which may reduce the effectiveness of vaccines have been recommended for patients with IBD [13,14]. 

BNT162b2 is a nucleoside-modified RNA vaccine encoded by the SARS-CoV-2 receptor-binding domain [15]. IBD patients were excluded from the BNT162b2 clinical trials [2]; therefore, real-world data and some prospective studies have recently been reported in this population [16,17,18,19]. However, only one study was conducted to evaluate the immunogenicity in pediatric IBD patients, which compared those treated with infliximab and vedolizumab (gut-selective anti-integrin α4ß7 monoclonal antibody), excluding those who were taking other immunosuppressive drugs [19]. Furthermore, SARS-CoV-2 is constantly mutating, indicating that it continuously evolves to develop an escape mechanism against vaccines or innate immunity, as demonstrated by the recent globally dominant variant Omicron, which was first identified in Botswana and South Africa [20]. 

Therefore, we hypothesized that immunosuppressed drug use and circulating new variants would reduce vaccine immunogenicity and efficacy in this population. We aimed to evaluate the immunogenicity of BNT162b2 in patients with IBDs relative to controls and assess the efficacy of vaccination against the Omicron variant in pediatric patients with IBD.

## 2. Materials and Methods

### 2.1. Study Design and Participants 

We conducted a prospective multicenter case–control study to compare the immunogenicity, safety, and efficacy of the anti-SARS-CoV-2 mRNA vaccine BNT162b2 (Comirnaty, Pfizer–BioNTech, USA) in patients with IBD and controls from November 2021 to April 2022 in Korea. There were 38 participants (control = 15 and IBD = 23) enrolled in this study who received COVID-19 vaccination. The IBD group included children diagnosed with CD or UC who were currently treated with IBD treatment drugs. The control groups included volunteers who visited clinics for health examination after recovering from acute illness or the routine follow-up of stable underlying disease not related to immunosuppression (i.e., nonalcoholic fatty liver disease and long-term follow-up after Kawasaki disease). 

We collected demographic data including age, sex, height, weight, underlying diseases, and medical history of SARS-CoV-2 infection of our participants. For patients with IBD, we also collected the results of laboratory tests, such as hemoglobin, erythrocyte sedimentation rate (ESR), stool calprotectin, the current status of medication, and IBD activity index scores, such as the pediatric Crohn’s disease activity index (PCDAI) and pediatric ulcerative colitis activity index (PUCAI) at each visit. Active disease was defined as PCDAI or PUCAI ≥ 10 in both CD and UC patients [21,22,23]. We classified IBD medications into four subgroups: 5-aminosalicylic acid (5-ASA) monotherapy, immunomodulator monotherapy (including azathioprine or MTX), anti-TNF (infliximab) monotherapy, and combination therapy (immunomodulatory + anti-TNF). None of the participants received anti-TNF monotherapy. 

### 2.2. Neutralizing Antibody Response Assessment

The participants were immunized at a government-qualified vaccine center for COVID-19. In Korea, all vaccine centers for COVID-19 are under quality control by the government through education programs of clinicians, thermal record monitoring for cold chain vaccine delivery, and an online vaccine registration system. Immunization records of participants included the date, time, injector, lot number of vaccines, and the name of the institution, data were verified through the online immunization record system of the Korea Disease Control and Prevention Agency. 

Serum samples were collected to assess humoral responses at two-time points: before the first dose of vaccination and within three months after the second dose of vaccination. To assess the potential neutralizing capacity of patient sera, the cPassTM SARS-CoV-2 Neutralization Antibody Detection Kit (GenScript Biotech, Mainz, Germany) was used. This CE/IVD-certified surrogate virus neutralization test (sVNT) quantifies the inhibition of receptor binding domain protein binding to the human host cell receptor protein ACE2 by patient antibodies in a blocking ELISA format and has been shown to correlate well with virus neutralization assays [24,25]. Samples were diluted tenfold and measured in duplicate, according to the manufacturer’s instructions. Inhibition (%) was calculated as follows: (1−OD value of sample/OD value of negative control) × 100. Sample values below the threshold of 30% were considered negative; values at or above the cutoff indicated the presence of SARS-CoV-2 neutralizing antibodies. At this cutoff, the negative and positive percentage agreement with the conventional plaque reduction neutralization test (PRNT)50 and PRNT90 assays was approximately 100%. The manufacturer reported the sensitivity and specificity for the assay to be 93.80% and 99.4%, respectively. As per kit specifications, patients with neutralization levels below 30% were considered negative for neutralizing antibodies. 

### 2.3. Assessment of Safety and Breakthrough Infection

Safety assessments included monitoring through a participant-directed vaccine diary for solicited local and systemic adverse events for seven days after each dose. Participants were also requested to report any unsolicited adverse events until the second visit on a diary card. Adverse events were categorized by the Food and Drug Administration toxicity grading scale for volunteers in vaccine trials as follows: grade 1 (no interference in daily activities), grade 2 (some interference in daily activities), grade 3 (participants unable to perform daily activities), and grade 4 (potentially life-threatening) [26]. 

The incidence rate of COVID-19 in our participants was also evaluated for efficacy of the vaccine because the Omicron variant was the dominant strain in Korea during the study period. We collected any additional information on the breakthrough SARS-CoV-2 infection rate through phone calls after approximately two months of BNT162b2 vaccination. Participants who were vaccinated 180 days before the peak of the Omicron outbreak were excluded from this analysis to eliminate confounding factors (waning of immunity) other than differences between groups. We enquired about the history of SARS-CoV-2 infection and the severity of the disease, such as the duration of fever, hospitalization, and need for oxygen support. Moreover, we collected data on delayed adverse reactions to the BNT162b2 vaccine. This phone call was performed with the parents’ informed consent and was conducted from the 22 to 30 April.

### 2.4. Statistical Analysis

We used descriptive statistics for the demographic data and safety profiles. All data are expressed as the mean ± standard deviation or median with interquartile range (IQR) according to their distribution. The chi-squared test with Fisher’s exact test was used to compare categorical variables such as sex and adverse effects. Pre- and post-vaccination titers were compared using the Mann–Whitney U test between the control and IBD groups. The Wilcoxon rank-sum test was used to compare post-vaccination titers among the IBD drugs. The Kaplan–Meier survival curves were used to compare the remaining subjects without breakthrough infections in the two groups during the investigation period, which was the Omicron-variant-dominant pandemic [27,28]. All statistical analyses were performed using SPSS version 29.0 (Chicago, IL, USA) and GraphPad Prism version 9.1.1 (GraphPad Software, San Diego, CA, USA). Differences were considered statistically significant with two-sided *p*-values < 0.05. 

## 3. Results

### 3.1. Study Population

Among all 38 participants, 23 patients with IBD had CD (*n*= 16) or UC (*n* = 7). Fifteen controls were healthy (*n* = 3) or had some resolved medical issues, including those not influencing their immunogenicity (fatty liver (*n* = 7), a history of Kawasaki disease (*n* = 2), thyroid disease (*n* = 1), history of histiocytic necrotizing lymphadenopathy (*n* = 1), and cardiac murmur (*n* = 1). None of the controls received immunosuppressive agents or had acute illnesses related to immunosuppressed conditions during the study period.

The mean age was 15.0 ± 1.5 (control group 14.5 ± 1.7, IBD group 15.2 ± 1.3) years; and 16 (69.6%) were males with IBD, and 11 (73.3%) were in the control group. There were no statistical differences in age and sex between the two groups (*p* = 1.0 and *p* = 0.299, respectively). Only one IBD participant had a previous SARS-CoV-2 infection (during the Delta-variant-dominant period) at the time of the study enrollment. 

Median sampling days after two doses of vaccine was 24.5 (IQR 21.0–30.3) days. There were no significant differences in median sampling date between the control (median 25, IQR 21–32) and IBD (median 24, IQR 20–30) groups (*p* = 0.697). We could not collect pre-vaccination serum from nine patients; instead, we confirmed that they had no history of COVID-19 before vaccination. 

Most of our IBD patients (*n* = 22, 95.7%) had received immunomodulators such as AZP (*n* = 10) or MTX (*n* = 5) at the time of enrollment. Almost 40% of the patients received combination therapy with anti-TNF drugs and other immunomodulators (Table 1). Eight (34.9%) patients with IBD were in active disease at the time of enrollment in the study, and five (21.7 %) remained active at the post-vaccination sampling period. After two doses of the vaccine, one patient with CD changed their therapy from MTX monotherapy to combination therapy with anti-TNF + MTX. 

### 3.2. sVNT Inhibition Levels of Anti-SARS-CoV-2 Antibody Level after Two Doses of BNT162b2

**Figure 1:**
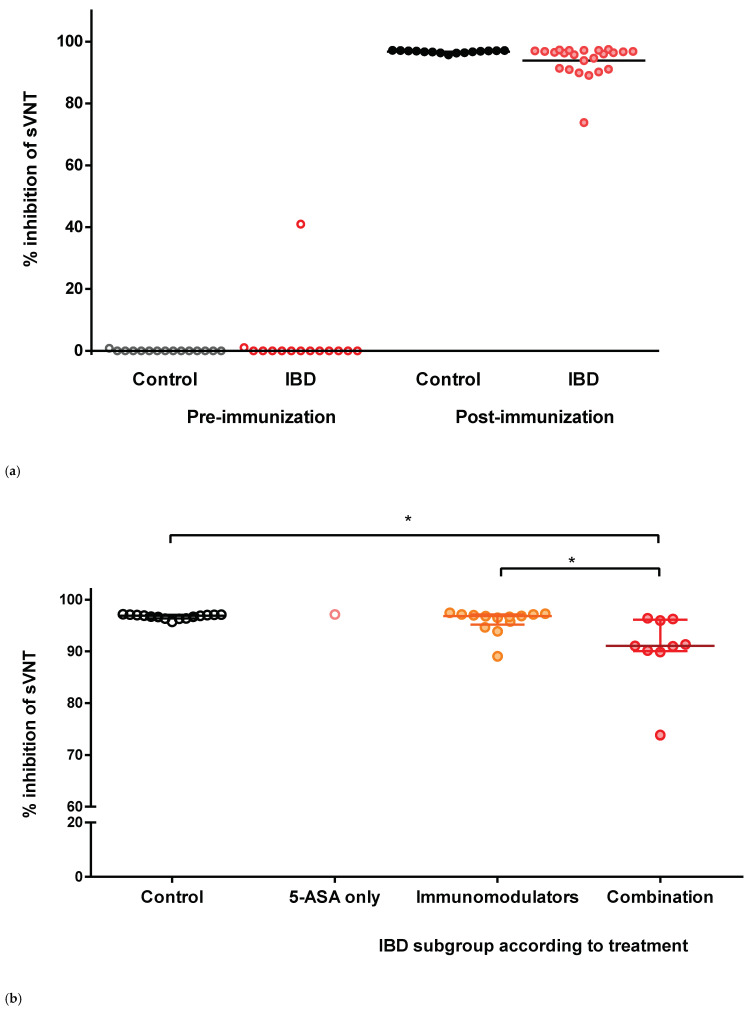

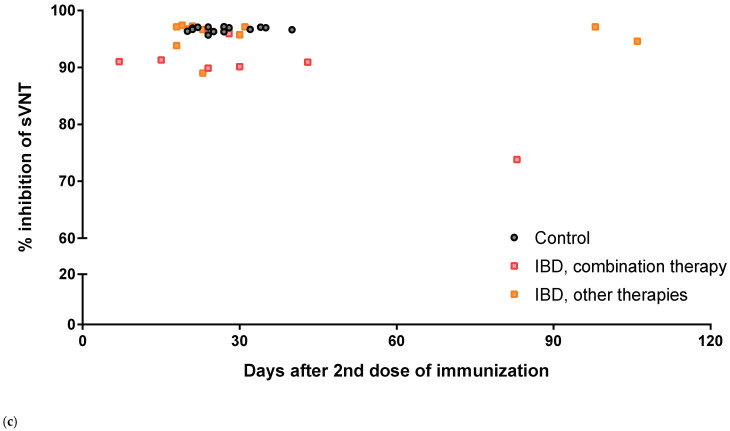
Scatter plot for the percentage inhibition of sVNT. (**a**) Total participants. (**b**) Treatment drugs before immunization. (**c**) According to days after the 2nd dose of immunization at sampling. * indicates *p* = 0.002.

The neutralizing capacities of participants’ sera against the receptor-binding domain of SARS-CoV-2 were measured by sVNT. The median level of percentage inhibition before vaccination was 0 in both the control and IBD groups. Only one patient with IBD had a pre-vaccination level of 41% and had a history of COVID-19. After two doses of vaccine, the median level of percentage inhibition was significantly increased (median 96.7, IQR 95.4–97.0): control group, 96.9 (IQR 96.4–97.1); and IBD group, 96.3 (IQR 91.0–97.0), respectively (Figure 1a). There were no significant difference in the post-vaccination sVNT levels between the two groups (*p* = 0.066).

Furthermore, we did not find any differences in the inhibition rate according to active IBD or the type of IBD (CD vs. UC). The median pre-vaccination levels in patients with active/non-active IBD and CD/UC were 0 (*p* = 0.917 and *p* = 0.584, respectively). The median inhibition levels after two doses of the vaccine were also not significantly different between patients with active IBD (median 96.5, IQR 94.5–96.9) and non-active IBD (median 95.7, IQR 90.2–97.1, *p* = 0.333). There was no difference in the inhibition level between CD (median 96.26, IQR 91.3–96.7) and UC (median 97.0, IQR 96.1–97.2, *p* = 0.88). 

To evaluate the impact of IBD medication on immunogenicity, the percentage inhibition of the treatment subgroups was compared. There were no significant differences among the IBD medication subgroups, except for combination therapy (Figure 1b). The percentage inhibition of the combination therapy subgroup was significantly reduced compared with that of the control group (*p* = 0.002) and immunomodulator monotherapy subgroup (*p* = 0.002, Figure 1b). 

Most of our participants had collected their samples between 2 and 7 weeks after the second dose of vaccine, and three patients with IBD were collected relatively late, after approximately 83 to 106 days (Figure 1c). Among them, CD patients with combination therapy showed a relatively low level of percentage inhibition (74%) 83 days after immunization, whereas the other two patients with IBD maintained high percentage inhibition at days 98 (97%) and 106 (95%). No significant negative correlation was observed between the level of percentage inhibition and duration after the completion of vaccination (*p* = 0.614).

Each dot represents the level of percentage inhibition in each subject’s serum. Horizontal bars indicate the median and interquartile ranges. sVNT, surrogate virus neutralization test; IBDs, inflammatory bowel diseases; 5-ASA, 5-aminosalicylic acid.

### 3.3. Safety Profiles

Data for solicited adverse events (AEs) were collected seven days after immunization, and those for unsolicited or serious AEs were collected until the second visit. A total of 27 participants submitted their diaries (Table 2). Serious AEs such as hospitalization or death were not reported. Acute myocarditis, a serious AE in this age group, was not reported in our study, although a mild degree of chest pain was reported in three cases. Pain at the injection site was the most frequent AE in both groups at both doses. In the control group, solicited AEs were reported in 80% of the participants. However, these symptoms were relatively low-grade (under grade 4), and all participants fully recovered within five days. Unsolicited AEs, including cervical lymphadenopathy (*n* = 1) and chest pain (*n* = 3), were reported, and the affected participants also recovered. Compared with the control group, fewer participants with IBD experienced AEs after the second dose of immunization. Systemic AEs, including fever and headache, were frequently reported in both groups. Although the difference was not statistically significant, the incidence rate of AEs was higher after the second dose of immunization. However, the IBD group did not show a significant increase in the rate of AE after a second dose of the vaccine, and the incidence of headache and fever was significantly lower than that in the control group.

Furthermore, when participants were asked about the long-term adverse effects of the vaccine after two months of immunization, children who received a phone call recalled no remarkable AEs.

### 3.4. Efficacy and Breakthrough Infection during the Omicron Variant Outbreak

**Figure 2:**
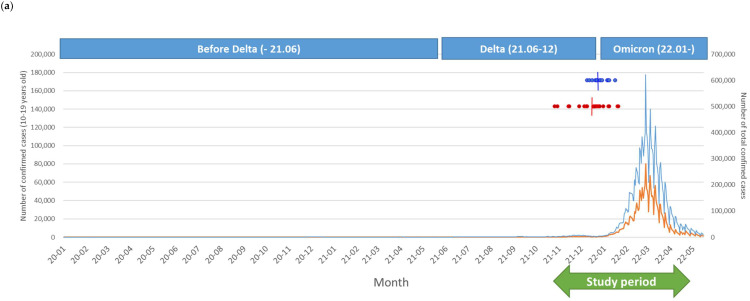

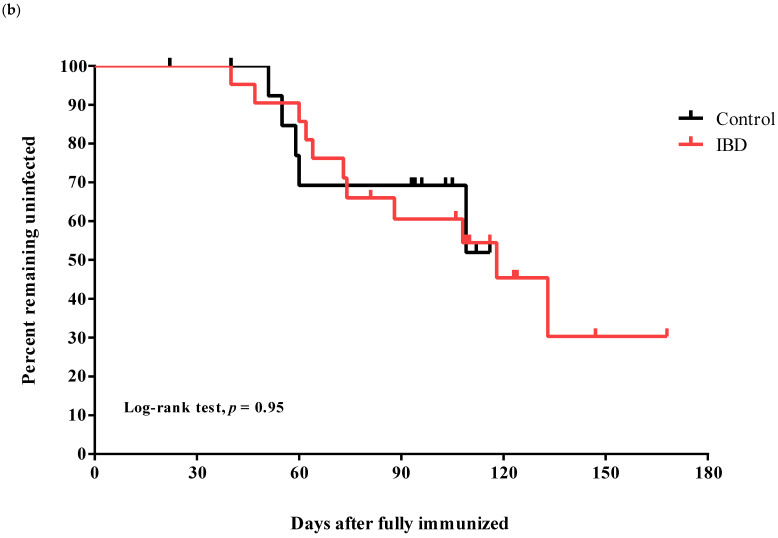
Breakthrough infection during the investigational period. (**a**) Number of confirmed coronavirus disease 2019 cases and dominant variants in Korea and our study population. The blue line indicates total cases, and the orange line indicates the number of adolescent cases (10–19 years of age) in the Korean population. Dark blue dots indicate the date of the second dose of immunization for the control group, and red dots indicate the IBD group in our study. Short vertical lines show the median date of the second immunization in each group. (**b**) Kaplan–Meier curves for the infection-free survival from breakthrough infections in the two groups during the investigation period coincided with those during the Omicron outbreak.

The Omicron strain was the dominant variant following the complete immunization of our participants, and the peak incidence of the Omicron outbreak was reported on 17 March 2022 (Figure 2a). Thirty-six participants were vaccinated at least 180 days prior to the Omicron outbreak. There was no significant difference between the two groups in the period from the second dose of immunization to the date of peak incidence of the Omicron variant outbreak. During follow-up, breakthrough infections of COVID-19 were observed in 16 cases (44.4%), without significant differences between the two groups: control (*n* = 5, 33.3%) and IBD (*n* = 11, 52.3%, *p* = 0.32). Breakthrough infection rates were not different among the IBD medication subgroups or those with active disease status: immunomodulator monotherapy (*n* = 6, 50.0%) vs. combination therapy (*n* = 5, 62.5%, *p* = 0.67); and active disease (*n* = 5, 62.5%) vs. inactive disease (*n* = 6, 46.2%, *p* = 0.183). Kaplan–Meier curves for COVID-19-free survival for the subjects are shown in Figure 2b. Although the total observational periods were different between the groups, the COVID-19-free survival curves were not significantly different (log-rank: *p* = 0.95). 

## 4. Discussion

This was a prospective case–control study of pediatric IBD patients in Korea to compare the neutralizing antibodies and efficacy of the COVID-19 vaccine during the Omicron outbreak. Although some studies have been conducted on adult IBD patients regarding the immunogenicity of COVID-19 vaccination and natural serologic response, only a few studies have been conducted in pediatric IBD patients, and there has been no study of BNT162b2 vaccine immunogenicity among Korean pediatric IBD patients. Vaccination responses differ according to age, ethnicity, nutritional status, and immune response [29,30]; therefore, we conducted this prospective study to understand the effectiveness of the BNT162b2 vaccine in Korean children.

In our study, most of the participants achieved a sufficient neutralizing antibody status after two doses of the BNT162b2 vaccine (median 96.7, IQR 95.4–97.0), and no significant differences were observed between IBD patients and controls. In a previous retrospective study, the authors reported no significant differences in the immunogenicity of the BNT162b2 vaccine between juvenile idiopathic arthritis patients and healthy controls [31]. However, the neutralizing antibody responses were lower in IBD patients treated with anti-TNF plus immunomodulators. Immunomodulators can attenuate vaccination efficacy [13,32], as demonstrated in IBD patients treated with infliximab and/or immunomodulators who had a decreased immune response to hepatitis B, pneumococcal, and influenza vaccine [33,34,35]. Moreover, previous studies reported that infliximab and combination therapy with immunomodulators attenuated the serologic response to the SARS-CoV-2 vaccine [14,19,36], although a study on children did not show statistical differences among biologic regimens [37]. Regarding biological agents, a previous study conducted among patients receiving biological therapies showed a high seroconversion rate and neutralizing antibody levels after two doses of the SARS-CoV-2 vaccine. However, anti-TNF agents (infliximab and adalimumab) showed lower seroconversion rates and positive anti-SARS-CoV-2 neutralizing antibody rates than ustekinumab and vedolizumab: infliximab 74.5%/67.7%, adalimumab 81.7%/87.5%, ustekinumab 100%/92.3%, and vedolizumab 92.8%/92.8% [38]. Anti-TNF agents directly neutralize the pro-inflammatory cytokine (TNF-α), which could decrease T-cell-dependent antibody production, whereas vedolizumab is a gut-selective anti-integrin agent that has no systemic effect on the immune system [14,19,38]. However, the serologic response to SARS-CoV-2 infection is also lower and attenuated in patients receiving biologics, and immunization with COVID-19 showed a higher seroconversion rate than natural infection. Appropriate vaccination scheduling, including second doses, should be considered in this population [37,39]. Due to constant immune system activation, patients with IBD have an exhausted T-cell phenotype, which may affect memory T-cells involved in antibody production; therefore, IBD activity control is also important to achieve high immunogenicity [14].

This is the first report that analyzed the SARS-CoV-2 breakthrough infection rates among a pediatric population during the Omicron outbreak and found no differences between the IBD and control groups. However, the lack of statistical significance may be due to the small sample size. Although exhibiting sufficient neutralizing antibody titers, almost 50% of our participants were infected with SARS-CoV-2, suggesting a poor protective effect of BNT162b2 against the Omicron variant. In the United Kingdom, a large-scale study on vaccine effectiveness [40] revealed that two doses of ChAdOx1 nCoV-19 or BNT162b2 vaccine did not show a protective effect against symptomatic disease caused by the Omicron variant. Moreover, the vaccine effectiveness weaned over time: 65.5% 2–4 weeks after the second BNT162b2 dose, 15.4% after 15–19 weeks, and further to 8.8% after 25 or more weeks. However, no study has been conducted on IBD patients during an Omicron outbreak. There are only three retrospective studies evaluating breakthrough infection in IBD patients after two doses of the vaccine [18,41,42,43], and only one study included a pediatric population (0.4% of the study population) [41]. These studies also revealed no statistically significant difference in breakthrough infection rates between IBD patients (0.14–0.36%) and controls (0.1–0.3%), with a lower breakthrough infection rate than in our study [18,41,43].

In a previous study of 6078 patients with IBD, the severity of IBD was independently associated with COVID-19 outcomes, such as hospitalization and intensive care unit (ICU)/ventilation/death, especially in a young age group (age ≤ 50 years) [44]. In our study, 6 out of 11 patients with IBD had active disease, but none required hospitalization or ICU care because of COVID-19. However, we could not confirm the protective effect of BNT162b2 in preventing severe disease in this study because the Omicron variant rarely causes a severe disease that requires hospitalization [40]. However, we found that patients with IBD also had sufficient neutralizing antibodies compared with the control group, and no significant serious AEs were observed in either group. During the current pandemic caused by mild yet highly contagious variants, adherence to vaccination recommendations for immunocompromised populations requires the consideration of protection against infection, vaccine sustainability, safety, and the prevention of severe diseases. 

Our study had some limitations. First, we included a relatively small number of patients, and the subgroup analysis did not show significant statistical differences. Therefore, all results should be interpreted with caution, including the low rate of serious AEs. However, we found a significantly lower inhibition rate in IBD patients receiving combination therapy compared with the immunomodulator therapy and control groups. Considering that IBD is a rare disease and a prospective study in the pediatric population is difficult, this study provides valuable results, even with a small number of patients. Second, regarding Omicron outbreak analysis, we did not perform a SARS-CoV-2 confirmatory polymerase chain reaction (PCR) test, and there was a possibility of underestimating the breakthrough infection rate due to asymptomatic infections of SARS-CoV-2. However, the possibility of underestimation should have been relatively low because, during this period, all participants had to undergo weekly SARS-CoV-2 PCR tests to attend school.

To the best of our knowledge, no study has compared the immunogenicity of the BNT162b2 vaccine in pediatric patients with IBD according to their treatment with or without biologics. Moreover, there have been no pediatric studies using serologic data with simultaneous real-world breakthrough infections. This study demonstrates that the immunogenicity of BNT162b2 in pediatric patients with IBD was sufficient compared with that in the controls, although IBD patients with combination therapy showed relatively lower levels of neutralizing antibodies than immunomodulatory therapy or non-IBD controls. Moreover, we provided the efficacy of the BNT162b2 vaccine for preventing Omicron in children with and without IBD, which was deemed insufficient. However, there were no severe COVID-19 cases in our study. To determine the overall effectiveness of the vaccine, including the effect of reducing the severity of COVID-19, studies with a larger number of participants are needed. Although there was no difference in immunogenicity at one month, an analysis of antibody persistence is also required.

## 5. Conclusions

Immunization with BNT162b2 in adolescent IBD patients could induce neutralizing antibody responses against SARS-CoV-2 and safety profiles, similar to that of the control group. However, combination therapy with anti-TNF-and immunomodulators reduced the level of neutralizing antibodies. The efficacy of the BNT162b2 vaccine for preventing Omicron variant infection in children with and without IBD was insufficient.

## Figures and Tables

**Table 1:** Baseline characteristics of the subjects at the time of the vaccination.

Demographics	Controls (*n* = 15)	Patients with IBD (*n* = 23)
Age, mean ± SD (year)	14.7 ± 1.7	15.2 ± 1.3
Male sex, *n* (%)	11 (73.3)	16 (69.6)
Female, *n* (%)	4 (26.7)	7 (30.4)
History of COVID-19 before vaccination	0 (0.0)	1 (4.3)
Clinical characteristics of the IBD group		
Duration of IBD (months), mean ± SD		20.6 ± 15.9
CD, *n* (%)		16 (69.6%)
UC, *n* (%)		7 (30.4%)
Active disease *, CD, *n* (%)		6 (37.5%)
Active disease *, UC, *n* (%)		2 (28.6%)
Treatment, *n* (%)		
5-ASA monotherapy, *n* (%)		1 (4.3%)
Immunomodulator monotherapy, *n* (%)		13 (56.5%) AZP 10/MTX 3
Anti-TNF + immunomodulator, *n* (%)		9 (39.1%) AZP 7/MTX 2
Laboratory findings		
Hemoglobin (g/dL), mean ± SD		13.5 ± 1.5
Hematocrit (%), mean ± SD		40.1 ± 3.7
ESR (mm/h), median ± IQR		8 (3–11)
Albumin (g/dL), mean ± SD		4.7 ± 0.3
Calprotectin (ug/g), median ± IQR		801.0 (113.7–1131.3)

Abbreviations: IBD, inflammatory bowel disease; COVID-19, coronavirus disease 2019; SD, standard deviation; CD, Crohn’s disease; UC, ulcerative colitis; 5-ASA, 5-aminosalicylic acid; AZP, azathioprine; MTX, methotrexate; TNF, tumor necrosis factor; ESR, erythrocyte sedimentation rate; IQR, interquartile range. * Active disease was defined as pediatric Crohn’s disease activity index or pediatric ulcerative colitis activity index ≥ 10 in both CD and UC patients [21,22,23].

**Table 2:** Frequency of adverse events after each dose.

	Control		IBD	
	Dose 1 (*n* = 10)	Dose 2 (*n* = 10)	Dose 1 (*n* = 17)	Dose 2 (*n* = 17)
**Adverse events**	8 (80.0)	8 (80.0)	9 (52.9)	6 (47.1) *
**Local adverse events**				
Pain on injection site	8 (80.0)	7 (70.0)	8 (47.1)	6 (47.1)
Swelling	2 (20.0)	4 (40.0)	1 (5.9)	2 (11.8)
Redness	0 (0.0)	2 (20.0)	0 (0.0)	1 (5.9)
Nodularity	3 (30.0)	3 (30.0)	1 (5.9)	1 (5.9)
**Systemic adverse events**				
Fever	1 (10.0)	6 (60.0)	1 (5.9)	1 (5.9) *
Myalgia	2 (20.0)	4 (40.0)	3 (17.6)	4 (23.5)
Fatigue	4 (40.0)	6 (60.0)	5 (29.4)	5 (29.4)
Headache	0 (0.0)	7 (70.0)	2 (11.8)	3 (17.6) *
Skin rash	0 (0.0)	1 (10.0)	1 (5.9)	1 (5.9)
Other	Cervical lymphadenopathy	Chest pain	Chest pain	Chest pain
**Serious adverse events**	0 (0.0)	0 (0.0)	0 (0.0)	0 (0.0)

Data are shown as the number and percentage, *n* (%). * *p* < 0.05 compared between groups.

## Data Availability

Not applicable.

## References

[1] Chen N., Zhou M., Dong X., Qu J., Gong F., Han Y., Qiu Y., Wang J., Liu Y., Wei Y. (2020). Epidemiological and clinical characteristics of 99 cases of 2019 novel coronavirus pneumonia in Wuhan, China: A descriptive study. Lancet.

[2] Polack F.P., Thomas S.J., Kitchin N., Absalon J., Gurtman A., Lockhart S., Perez J.L., Pérez Marc G., Moreira E.D., Zerbini C. (2020). Safety and efficacy of the BNT162b2 mRNA COVID-19 vaccine. N. Engl. J. Med..

[3] JCVI Statement on Vaccination of Children Aged 5 to 11 Years Old. GOV.UK. https://www.gov.uk/government/publications/jcvi-update-on-advice-for-covid-19-vaccination-of-children-aged-5-to-11/jcvi-statement-on-vaccination-of-children-aged-5-to-11-years-old.

[4] Committee on Infectious Diseases (2021). COVID-19 vaccines in children and adolescents. Pediatrics.

[5] European Centre for Disease Prevention and Control. https://www.ecdc.europa.eu/en/publications-data/interim-public-health-considerations-covid-19-vaccination-children-aged-5-11.

[6] Kim B.J., Song S.M., Kim K.M., Lee Y.J., Rhee K.W., Jang J.Y., Park S.J., Yoon C.H. (2010). Characteristics and trends in the incidence of inflammatory bowel disease in Korean children: A single-center experience. Dig. Dis. Sci..

[7] Ng W.K., Wong S.H., Ng S.C. (2016). Changing epidemiological trends of inflammatory bowel disease in Asia. Intest. Res..

[8] Rosen M.J., Dhawan A., Saeed S.A. (2015). Inflammatory bowel disease in children and adolescents. JAMA Pediatr..

[9] Turner D., Ruemmele F.M., Orlanski-Meyer E., Griffiths A.M., de Carpi J.M., Bronsky J., Veres G., Aloi M., Strisciuglio C., Braegger C.P. (2018). Management of paediatric ulcerative colitis, Part 1: Ambulatory care-an evidence-based guideline from European Crohn’s and Colitis Organization and European Society of Paediatric Gastroenterology, hepatology and nutrition. J. Pediatr. Gastroenterol. Nutr..

[10] Van Rheenen P.F., Aloi M., Assa A., Bronsky J., Escher J.C., Fagerberg U.L., Gasparetto M., Gerasimidis K., Griffiths A., Henderson P. (2020). The medical management of paediatric Crohn’s disease: An ECCO-ESPGHAN guideline update. J. Crohn’s Colitis.

[11] Beaugerie L., Kirchgesner J. (2019). Balancing benefit vs. risk of immunosuppressive therapy for individual patients with inflammatory bowel diseases. Clin. Gastroenterol. Hepatol..

[12] Rotondo J.C., Bononi I., Puozzo A., Govoni M., Foschi V., Lanza G., Gafà R., Gaboriaud P., Touzé F.A., Selvatici R. (2017). Merkel cell carcinomas arising in autoimmune disease affected patients treated with biologic drugs, including anti-TNF. Clin. Cancer Res..

[13] Alexander J.L., Moran G.W., Gaya D.R., Raine T., Hart A., Kennedy N.A., Lindsay J.O., MacDonald J., Segal J.P., Sebastian S. (2021). SARS-CoV-2 vaccination for patients with inflammatory bowel disease: A British Society of Gastroenterology inflammatory bowel Disease section and IBD Clinical Research Group position statement. Lancet Gastroenterol. Hepatol..

[14] Doherty J., Fennessy S., Stack R., O’Morain N., Cullen G., Ryan E.J., De Gascun C., Doherty G.A. (2021). Review Article: Vaccination for patients with inflammatory bowel disease during the COVID-19 pandemic. Aliment. Pharmacol. Ther..

[15] Walsh E.E., Frenck R.W., Falsey A.R., Kitchin N., Absalon J., Gurtman A., Lockhart S., Neuzil K., Mulligan M.J., Bailey R. (2020). Safety and immunogenicity of two RNA-based COVID-19 vaccine candidates. N. Engl. J. Med..

[16] Hadi Y.B., Thakkar S., Shah-Khan S.M., Hutson W., Sarwari A., Singh S. (2021). COVID-19 vaccination is safe and effective in patients with inflammatory bowel disease: Analysis of a Large Multi-institutional Research Network in The United States. Gastroenterology.

[17] Widdifield J., Kwong J.C., Chen S., Eder L., Benchimol E.I., Kaplan G.G., Hitchon C., Aviña-Zubieta J.A., Lacaille D., Chung H. (2022). Vaccine effectiveness against SARS-CoV-2 infection and severe outcomes among individuals with immune-mediated inflammatory diseases tested between March 1 and Nov 22, 2021, in Ontario, Canada: A population-based analysis. Lancet Rheumatol..

[18] Ben-Tov A., Banon T., Chodick G., Kariv R., Assa A., Gazit S., Collaborators of the Maccabi Institute for Research & Innovation COVID-19 Task Force (2021). BNT162b2 messenger RNA COVID-19 vaccine effectiveness in patients with inflammatory bowel disease: Preliminary real-world data during mass vaccination campaign. Gastroenterology.

[19] Kennedy N.A., Lin S., Goodhand J.R., Chanchlani N., Hamilton B., Bewshea C., Nice R., Chee D., Cummings J.F., Fraser A. (2021). Infliximab is associated with attenuated immunogenicity to BNT162b2 and ChAdOx1 nCoV-19 SARS-CoV-2 vaccines in patients with IBD. Gut.

[20] World Health Organization. https://www.who.int/news/item/26-11-2021-classification-of-Omicron-(b.1.1.529)-sars-cov-2-variant-of-concern.

[21] Turner D., Otley A.R., Mack D., Hyams J., de Bruijne J., Uusoue K., Walters T.D., Zachos M., Mamula P., Beaton D.E. (2007). Development, validation, and evaluation of a pediatric ulcerative colitis activity index: A prospective multicenter study. Gastroenterology.

[22] Hyams J.S., Ferry G.D., Mandel F.S., Gryboski J.D., Kibort P.M., Kirschner B.S., Griffiths A.M., Katz A.J., Grand R.J., Boyle J.T. (1991). Development and validation of a pediatric Crohn’s disease activity index. J. Pediatr. Gastroenterol. Nutr..

[23] Hyams J., Markowitz J., Otley A., Rosh J., Mack D., Bousvaros A., Kugathasan S., Pfefferkorn M., Tolia V., Evans J. (2005). Evaluation of the pediatric Crohn disease activity index: A prospective multicenter experience. J. Pediatr. Gastroenterol. Nutr..

[24] von Rhein C., Scholz T., Henss L., Kronstein-Wiedemann R., Schwarz T., Rodionov R.N., Corman V.M., Tonn T., Schnierle B.S. (2021). Comparison of potency assays to assess SARS-CoV-2 neutralizing antibody capacity in COVID-19 convalescent plasma. J. Virol. Methods.

[25] Tan C.W., Chia W.N., Qin X., Liu P., Chen M.I., Tiu C., Hu Z., Chen V.C., Young B.E., Sia W.R. (2020). A SARS-CoV-2 surrogate virus neutralization test based on antibody-mediated blockage of ACE2-spike protein-protein interaction. Nat. Biotechnol..

[26] Food and Drug Administration. https://www.fda.gov/regulatory-information/search-fda-guidance-documents/toxicity-grading-scale-healthy-adult-and-adolescent-volunteers-enrolled-preventive-vaccine-clinical.

[27] Calabuig J.M., García-Raffi L.M., García-Valiente A., Sánchez-Pérez E.A. (2021). Kaplan-Meier type survival curves for COVID-19: A health data based decision-making tool. Front. Public Health.

[28] Sormani M.P., Schiavetti I., Inglese M., Carmisciano L., Laroni A., Lapucci C., Visconti V., Serrati C., Gandoglia I., Tassinari T. (2022). Breakthrough SARS-CoV-2 infections after COVID-19 mRNA vaccination in MS patients on disease modifying therapies during the Delta and the Omicron waves in Italy. EBioMedicine.

[29] Abu Jabal K., Ben-Amram H., Beiruti K., Batheesh Y., Sussan C., Zarka S., Edelstein M. (2021). Impact of age, ethnicity, sex and prior infection status on immunogenicity following a single dose of the BNT162b2 mRNA COVID-19 vaccine: Real-world evidence from healthcare workers, Israel, December 2020 to January 2021. EuroSurveill..

[30] Falahi S., Kenarkoohi A. (2022). Hosts. Host factors and vaccine efficacy: Implications for COVID-19 vaccines. J. Med. Virol..

[31] Gicchino M.F., Abbate F.G., Amodio A., Miraglia del Giudice E., Olivieri A.N. (2022). Preliminary observations on the immunogenicity and safety of vaccines to prevent COVID-19 in patients with juvenile idiopathic arthritis. Acta Paediatr..

[32] Papp K.A., Haraoui B., Kumar D., Marshall J.K., Bissonnette R., Bitton A., Bressler B., Gooderham M., Ho V., Jamal S. (2019). Vaccination guidelines for patients with immune-mediated disorders on immunosuppressive therapies. J. Cutan. Med. Surg..

[33] Andrade P., Santos-Antunes J., Rodrigues S., Lopes S., Macedo G. (2015). Treatment with infliximab or azathioprine negatively impact the efficacy of hepatitis B vaccine in inflammatory bowel disease patients. J. Gastroenterol. Hepatol..

[34] Hagihara Y., Ohfuji S., Watanabe K., Yamagami H., Fukushima W., Maeda K., Kamata N., Sogawa M., Shiba M., Tanigawa T. (2014). Infliximab and/or immunomodulators inhibit immune responses to trivalent influenza vaccination in adults with inflammatory bowel disease. J. Crohn’s Colitis.

[35] Fiorino G., Peyrin-Biroulet L., Naccarato P., Szabò H., Sociale O.R., Vetrano S., Fries W., Montanelli A., Repici A., Malesci A. (2012). Effects of immunosuppression on immune response to pneumococcal vaccine in inflammatory bowel disease: A prospective study. Inflam. Bowel Dis..

[36] Alexander J.L., Kennedy N.A., Ibraheim H., Anandabaskaran S., Saifuddin A., Castro Seoane R., Liu Z., Nice R., Bewshea C., D’Mello A. (2022). COVID-19 vaccine-induced antibody responses in immunosuppressed patients with inflammatory bowel disease (VIP): A multicentre, prospective, case-control study. Lancet Gastroenterol. Hepatol..

[37] Dailey J., Kozhaya L., Dogan M., Hopkins D., Lapin B., Herbst K., Brimacombe M., Grandonico K., Karabacak F., Schreiber J. (2022). Antibody responses to SARS-CoV-2 after infection or vaccination in children and young adults with inflammatory bowel disease. Inflam. Bowel Dis..

[38] Shehab M., Alrashed F., Alfadhli A., Alotaibi K., Alsahli A., Mohammad H., Cherian P., Al-Khairi I., Alphonse Thanaraj T., Channanath A. (2021). Serological response to BNT162b2 and ChAdOx1 nCoV-19 vaccines in patients with inflammatory bowel disease on biologic therapies. Vaccines.

[39] Kennedy N.A., Goodhand J.R., Bewshea C., Nice R., Chee D., Lin S., Chanchlani N., Butterworth J., Cooney R., Croft N.M. (2021). Anti-SARS-CoV-2 antibody responses are attenuated in patients with IBD treated with infliximab. Gut.

[40] Andrews N., Stowe J., Kirsebom F., Toffa S., Rickeard T., Gallagher E., Gower C., Kall M., Groves N., O’Connell A.M. (2022). COVID-19 vaccine effectiveness against the Omicron (B.1.1.529) variant. N. Engl. J. Med..

[41] Lev-Tzion R., Focht G., Lujan R., Mendelovici A., Friss C., Greenfeld S., Kariv R., Ben-Tov A., Matz E., Nevo D. (2022). COVID-19 vaccine is effective in inflammatory bowel disease patients and is not associated with disease exacerbation. Clin. Gastroenterol. Hepatol..

[42] Khan N., Mahmud N. (2021). Effectiveness of SARS-CoV-2 vaccination in a Veterans Affairs cohort of patients with inflammatory bowel disease with diverse exposure to immunosuppressive medications. Gastroenterology.

[43] Bhurwal A., Mutneja H., Bansal V., Goel A., Arora S., Attar B., Minacapelli C.D., Kochhar G., Chen L.A., Brant S. (2022). Effectiveness and safety of SARS-CoV-2 vaccine in inflammatory bowel Disease patients: A systematic review, meta-analysis and meta-regression. Aliment. Pharmacol. Ther..

[44] Ricciuto A., Lamb C.A., Benchimol E.I., Walker G.J., Kennedy N.A., Kuenzig M.E., Kaplan G.G., Kappelman M.D., Ungaro R.C., Colombel J.F. (2022). Inflammatory bowel disease clinical activity is associated with COVID-19 severity especially in younger patients. J. Crohn’s Colitis.

